# Monomeric, porous type II collagen scaffolds promote chondrogenic differentiation of human bone marrow mesenchymal stem cells *in vitro*

**DOI:** 10.1038/srep43519

**Published:** 2017-03-03

**Authors:** M. Tamaddon, M. Burrows, S. A. Ferreira, F. Dazzi, J. F. Apperley, A. Bradshaw, D. D. Brand, J. Czernuszka, E. Gentleman

**Affiliations:** 1 Department of Materials, University of Oxford, Oxford OX1 3PH, UK; 2 Craniofacial Development and Stem Cell Biology, King’s College London, London SE1 9RT, UK; 3 Division of Cancer Studies, Rayne Institute, King’s College London, London SE5 9NU, UK; 4 Centre for Haematology, Department of Medicine, Imperial College London, London W12 0NN, UK; 5 John Goldman Centre for Cellular Therapy, Imperial College Healthcare NHS Trust, London W12 0HS, UK; 6 Research Service, Memphis VA Medical Center, Departments of Medicine and Microbiology, Immunology and Biochemistry, University of Tennessee Health Science Center, Memphis, TN 38104, USA

## Abstract

Osteoarthritis (OA) is a common cause of pain and disability and is often associated with the degeneration of articular cartilage. Lesions to the articular surface, which are thought to progress to OA, have the potential to be repaired using tissue engineering strategies; however, it remains challenging to instruct cell differentiation within a scaffold to produce tissue with appropriate structural, chemical and mechanical properties. We aimed to address this by driving progenitor cells to adopt a chondrogenic phenotype through the tailoring of scaffold composition and physical properties. Monomeric type-I and type-II collagen scaffolds, which avoid potential immunogenicity associated with fibrillar collagens, were fabricated with and without chondroitin sulfate (CS) and their ability to stimulate the chondrogenic differentiation of human bone marrow-derived mesenchymal stem cells was assessed. Immunohistochemical analyses showed that cells produced abundant collagen type-II on type-II scaffolds and collagen type-I on type-I scaffolds. Gene expression analyses indicated that the addition of CS – which was released from scaffolds quickly – significantly upregulated expression of type II collagen, compared to type-I and pure type-II scaffolds. We conclude that collagen type-II and CS can be used to promote a more chondrogenic phenotype in the absence of growth factors, potentially providing an eventual therapy to prevent OA.

Osteoarthritis (OA) is a major cause of disability in the adult population^1^. Traumatic lesions to the cartilage surface are thought to progress to OA^2^. Therefore, early treatment may prevent OA progression and postpone the need for total joint replacement. Cartilage has a limited capacity to self-heal^3^, and current treatment options which include marrow stimulation (microfracture), mosaicplasty, and autologous chondrocyte implantation can often lead to the formation of inferior fibrocartilage instead of hyaline cartilage, and may be associated with donor site morbidity, pain, haematoma and inflammation^4^. Repairing cartilage using tissue engineering (TE) strategies may be an effective alternative^5^,^6^,^7^,^8^,^9^. TE approaches for cartilage repair provide a three-dimensional microenvironment for cell attachment, growth and differentiation, which can minimise chondrocyte dedifferentiation that occurs in two-dimensional cultures^10^. Nevertheless, the ideal scaffold with structural, chemical and mechanical properties that can direct appropriate progenitor cell differentiation and cartilage formation remains elusive.

Marrow stromal cells (often called mesenchymal stem cells, MSCs), can be derived from the bone marrow and have the ability to differentiate to chondrocytes (*e.g.* ref. 11) under appropriate biological, chemical or physical signals^12^. The composition of the extracellular matrix (ECM) on which stem cells grow has been shown to be particularly important in directing their differentiation^13^,^14^,^15^. That is, matrices specific to a particular tissue have been shown to drive stem cell differentiation down the same lineage. This has been one of the driving forces behind the development of decellularised matrices as scaffolds for TE. For example, it has been shown that MSCs grown on decellularised native bone ECM differentiated to osteoblasts without the addition of chemical factors (*e.g.* dexamethasone)^16^. Therefore, a potentially promising approach for cartilage TE is to mimic the composition and structure of the native ECM to fabricate a suitable scaffold. Articular cartilage is predominantly composed of collagen type II and the glycosaminoglycan chondroitin sulfate (CS)^3^. A scaffold that mimics this composition might effectively direct MSCs differentiation and may be suitable for cartilage regeneration.

Collagens for fabricating TE scaffolds are often extracted from tissues that are rich in the desired collagen type. Isolated collagen triple helices are “monomeric”, whereas extractions of collagen with a retained fibrillar structure are “polymeric”. Monomeric forms of collagen may be free of telopeptides (atelocollagen) or have the telopeptides intact (tropocollagen), whilst the polymeric form is insoluble and is composed of naturally cross-linked tropocollagen^17^. The majority of collagenous scaffolds^18^,^19^,^20^ for TE are fabricated using polymeric collagen due to the ease and low cost of its extraction. However, the presence of native crosslinks in polymeric collagen can induce batch-to-batch variability, reducing the reliability of the final product. Moreover, most collagens are extracted from animal tissues, which may introduce issues with immunogenicity^21^. Atelocollagens devoid of such immunogenic determinants and native crosslinks may be a preferred form of collagen for TE scaffolds. However, atelocollagens have only been used in a few studies^22^,^23^,^24^, so their suitability for TE is relatively unexplored. Atelocollagen can self-assemble *in vitro*, where fibril formation and fibril diameter will partly depend on the type of collagen^25^, method of extraction^26^ and precise processing conditions^27^. As the fibrils form, the molecular weight of the collagen increases, which may affect the subsequent collagen solution viscosity, and thus the mechanical stability and degradation properties of the final scaffold. Moreover, such changes may also affect the scaffold’s nanotopography^28^ (conferred by *d-banding* during fibrillogenesis), as well as its microstructural properties^29^, including pore size^30^, pore shape^31^, and compressive strength^32^,^33^, which may all influence cellular adhesion, morphology and differentiation.

CS is a polymeric carbohydrate with sulfated, negatively charged, repeating disaccharide units. CS is non-immunogenic and can affect cellular behaviours, including adhesion, migration, and differentiation^34^. The incorporation of CS into collagen fibrils stimulates the proliferation of chondrocytes *in vitro*^35^ whilst increasing cellular ingrowth and reducing foreign body response *in vivo*^36^. A number of studies have been conducted on type I and II collagen scaffolds into which CS was incorporated either by co-precipitation^18^,^19^,^37^,^38^ or covalent attachment of CS to the collagen^35^,^39^,^40^. Such collagen-CS matrices support viability and phenotype retention of chondrocytes, however, these matrices mainly contain insoluble collagens with their associated immunogenicity and batch-to-batch variability, and are often crosslinked by physical (*e.g.* dehydrothermal treatment (DHT)) or chemical (*e.g.*1-ethyl-3-(3-dimethylaminopropyl) carbodiimide/N-hydroxysuccinimide (EDC/NHS)) means, which may lead to partial denaturation of collagen or cytotoxicity^41^.

The aim of this study was to determine, if through tailoring of scaffold composition and physical properties we could promote progenitor cells to adopt more chondrogenic phenotypes. We have previously produced and characterised monomeric type II collagen–CS scaffolds^17^ and reported on their microstructure and mechanical properties. Here, we also produced atelocollagen afibrillar type I scaffolds and investigated the effect of collagen type and the incorporation of CS on cellular behaviour and their potential to direct stem cell differentiation in the absence of chemical induction or growth factors (Fig. 1). We show that afibrillar and reconstituted fibrillar type II collagen scaffolds containing CS support cellular viability and direct chondrogenic differentiation of human bone marrow mesenchymal stem cells (hBMMSCs).

## Results and Discussion

### Scaffold characterisation and microstructural evaluation

We fabricated afibrillar type I collagen scaffolds (1af) and afibrillar and reconstituted fibrillar type II collagen scaffolds (2af and 2rf, respectively) with and without CS (Table 1). Comparing characteristics of 1af scaffolds to those of our previously characterised type II scaffolds (Table 2-some data reproduced with permission for comparison^17^), many properties, including the compressive modulus of 1af scaffolds were comparable to that of 2af scaffolds. Scaffold stiffness may affect stem cell fate^32^ and porosity has been shown to regulate cellular behaviours^42^, including adhesion and ingrowth. Porosity is essential for diffusion of nutrients and gases to cells as well as the removal of metabolic waste. All of the produced scaffolds were over 99% porous.

**Figure 2:**
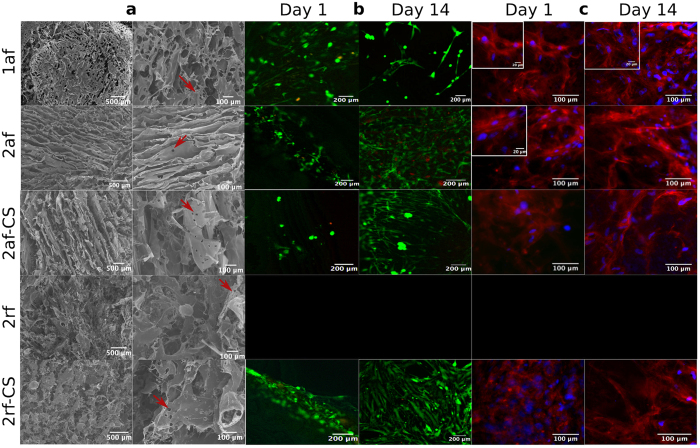
Scaffold microstructure and cell-scaffold interactions: (**a**) SEM micrographs showing the porous structure and internal features of collagen scaffolds. Red arrows indicate secondary pores, which are perforations in pore walls. (**b**) hBMMSCs viability by Live/Dead^®^ assay at day 1 and 14. Live cells are green and dead cells are red. (**c**) Actin cytoskeleton staining (red) and nuclei (blue) at day 1 and 14.

Scanning electron microscopy (SEM) images (Fig. 2a) of all groups showed that 1af scaffolds, like our previous finding on type II scaffolds, contained a microstructure of pores that visually appeared to be interconnected, with round or elongated pores depending on the precise composition. Scaffold pore size can affect cellular attachment, morphology and differentiation^30^. Pore shape, presented here as isoperimetric quotient of circularity (Q) (Table 2), may influence cellular fate. That is, progenitor cells have been shown to be directed towards a specific lineage by artificially controlling their shape. For example, BMMSCs with a more rounded cell shape have been associated with higher expression of chondrogenic markers^43^,^44^. Therefore, scaffolds with more circular pore shapes could be beneficial for chondrogenic differentiation. The distribution of elliptical pore areas in 1af scaffolds was significantly different from that in 2af (p = 0.00004), 2af-CS (p = 0.000004) and 2rf-CS (p = 0.00002) scaffolds. The axes sizes of elliptical pores were quantified to determine the proportion suitable to support cartilage tissue formation. For cartilage regeneration, pores of 50–300 μm are usually considered suitable for stimulating appropriate differentiation and tissue formation^45^,^46^. 2rf-CS scaffolds had the highest proportion of pores (38%) with both axes in an appropriate size range, followed closely by 2af-CS (31%) (Table 2). Based on this result, these two groups plus 2af were chosen for further cell analyses.

### Cell viability and metabolic activity on scaffolds

We hypothesised that a combination of the biological composition and physical properties of the scaffolds would direct hBMMSCs differentiation in the absence of additional chemical stimuli. To test this, we evaluated the ability of hBMMSCs to adhere, proliferate, and differentiate on type I and type II scaffolds. All scaffolds allowed for cell attachment and viability throughout 28 days. Live/Dead^®^ Cell viability assay (Fig. 2b) showed live cells (green) on all scaffold types with only a few dead cells (red). Immunofluorescence staining for actin showed a well-developed cytoskeleton suggesting cells were able to adopt spread morphologies on the scaffolds (Fig. 2c).

**Figure 3:**
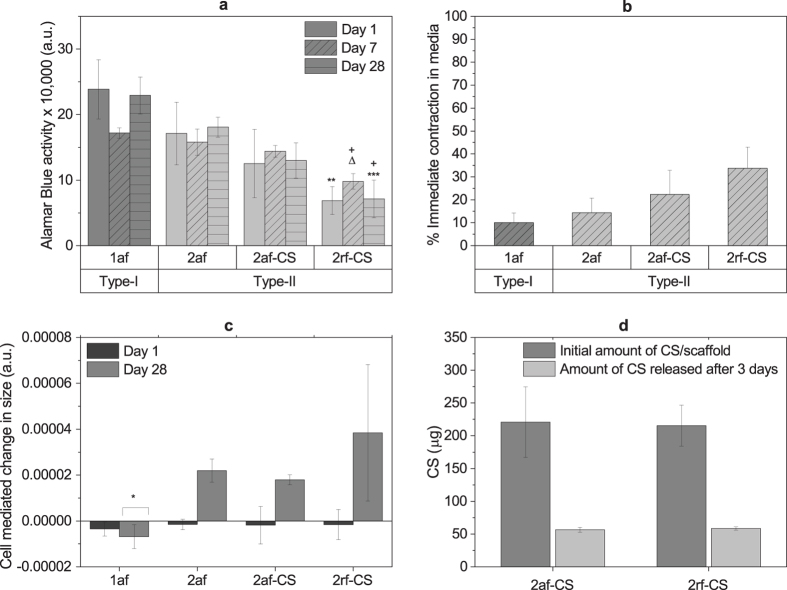
Cell proliferation, scaffold size changes and CS release: (**a**) Metabolic activity of cells on scaffolds determined by measuring alamarBlue activity (arbitrary unit: fluorescent intensity). One-way ANOVA followed by Tukey test was used to evaluate the alamarBlue data; *shows significantly lower metabolic activity compared to 1af at the same time point, *represents p = 0.05, **represents p = 0.005, *** represents p = 0.0003; + shows significantly lower metabolic activity compared to 2af at the same time point; ∆ significantly lower than 2af-CS, p = 0.002. (**b**) Changes in scaffold size upon contact with culture media. (**c**) Changes in scaffold size after 1 and 28 days in culture with cells (arbitrary unit: mm^2^/fluorescent activity). Whilst the size of type-I scaffolds decreased in the presence of cells, presumed matrix production mediated an increase in the size of type-II scaffolds. One-way ANOVA test with Fisher LSD was used to evaluate the scaffold size data; *shows significant difference compared to 2af (p = 0.02), 2af-CS (p = 0.03) and 2rf-CS (p = 0.004). (**d**) Initial amount of CS per dry scaffold and the amount released into cell culture media after seeding the scaffolds with cells.

AlamarBlue activity, a measure of cell metabolic activity, was quantified on day 1 as an indication of cell attachment. Cell attachment was higher on type-I scaffolds compared to type II scaffolds (2af-CS and 2rf-CS, p = 0.01 and p = 0.001, respectively) (Fig. 3a). This is believed to be partly related to the number of active cell binding sites on each; in type I collagen, the α1(I) chain of the triple helix is more active than the α1(II) chain in collagen type II, as it contains more active peptide residues^47^. After 7 days of culture, alamarBlue activity of cells on 2rf-CS scaffolds was significantly lower compared to that on all other compositions (1af, p = 0.0001; 2af, p = 0.0004; 2af-CS, p = 0.002) and the same trend was observed after 28 days, with lower activity on 2rf-CS than 1af (p = 0.00006) and 2af scaffolds (p = 0.0007).

### Modifications to scaffold physical properties and analyses of matrix synthesis

All scaffolds exhibited some degree of contraction when placed in cell culture medium (Fig. 3b). In order to assess the changes in scaffold size with culture and determine the cell-mediated and solution-mediated contributions, their sizes were monitored throughout 28 days. CS-containing scaffolds (2af-CS and 2rf-CS) showed the greatest decrease in size (25–35%) compared to pure scaffolds (1af and 2af) with 2rf-CS contracting significantly more when compared to pure scaffolds (1af, p = 0.01 and 2af, p = 0.02). Collagen scaffold shrinkage is widely recognised and may be problematic for TE. It can not only distort shape and dimensions when placed within a defect site, but also lead to constriction of pores affecting diffusive properties and cell migration^48^. Interestingly, after 28 days, cell-mediated size measurements, which we defined as changes in scaffold diameter measured in cell-containing scaffolds compared to the no cell controls, revealed an increase in type-II scaffold sizes that may have resulted from cell-mediated matrix deposition (Fig. 3c). This effect was not observed in type-I scaffolds (compared to 2af: p = 0.02, 2af-CS: p = 0.03 and 2rf-CS: p = 0.004).

To examine CS attachment and release from scaffolds, a total GAG assay was performed. Total GAG quantification showed that more than 25% of the CS incorporated in scaffolds (2af-CS and 2rf-CS) was released into the media in the first three days after immersion in cell culture media in the presence of cells (Fig. 3d). It is possible that the persistent exposure of the negatively charged CS to the positively charged amino acids in α-MEM media may have attracted them away from collagen causing its dispersion in the media. Indeed, the pronounced initial shrinkage of CS-containing scaffolds may have resulted, at least in part, from this loss of CS.

**Figure 4:**
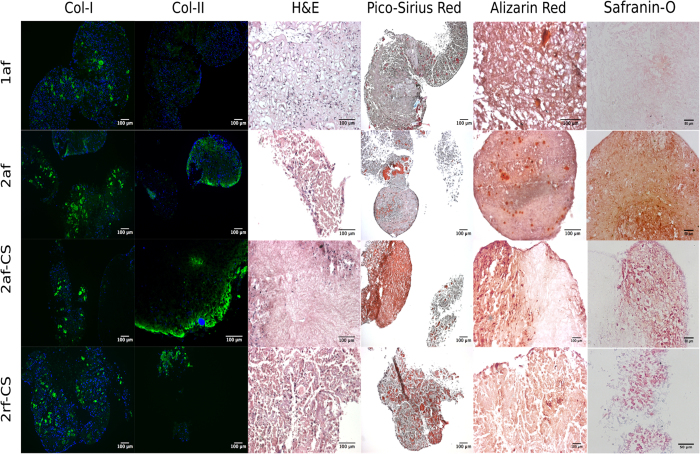
Histology and immunostaining of scaffolds: immunostaining of collagen type I and type-II on scaffolds after 28 days in culture, blue: nuclei and green: type I or II collagen. H&E, Picosirius Red, Safranin-O and Alizarin Red staining after 28 days in culture.

Histological examination of scaffolds after 28 days in culture (Fig. 4) showed that cell infiltration took place in all scaffolds, however, to a lesser extent in 2af-CS as evidenced by the sparsity of nuclear stain in H&E sections. Picosirius Red staining, an indicator of the presence of collagens, showed more staining in type-II scaffolds compared to type I, therefore, we speculate that a higher degree of collagen degradation took place in type I scaffolds compared to type II scaffolds or cells secreted more collagenous matrix when seeded on type II scaffolds. This is in keeping with scaffold size analyses, which showed an increase in scaffold diameter for type II scaffolds over the 28 day culture period. Alizarin Red staining was positive for calcium deposition on 1af and 2af scaffolds, but only background staining was evident on type II scaffolds that contained CS, suggesting that the lack of CS may have provided an environment suitable for osteogenesis. Positive staining for calcium could also have been mediated by the hypertrophic differentiation of hBMMSC’s. Further staining for markers of hypertrophic differentiation, such as type X collagen, would be necessary to rule out this possibility.

### Influence of collagen scaffold type on hBMMSC differentiation

Immunohistochemistry staining (Fig. 4) revealed type II collagen deposition by hBMMSCs on all type II scaffolds with minimal staining for type I collagen. Alternatively, on type-I scaffolds, we detected staining for human type I collagen throughout, with little to no staining for type II collagen. We also observed extensive staining for Safranin-O, an indirect indicator of proteoglycan deposition, on type-II scaffolds, but very little staining on type-I scaffolds (Fig. 4). These results suggest that the composition of the scaffold itself affected ECM secretion.

Differentiation of MSCs is governed by a network of signalling mechanisms, among which, SOX9 and RUNX2 are transcription factors expressed by MSCs upon their commitment towards chondrogenesis and osteogenesis, respectively^49^.

**Figure 5:**
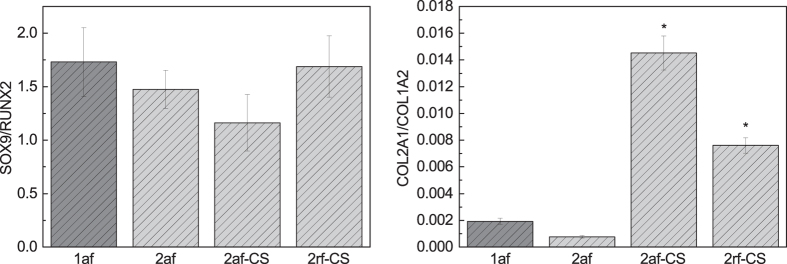
Assessment of cellular differentiation: relative expression of chondro- and osteogenic genes in hBMMSCs cultured on scaffolds for 28 days. One-way ANOVA test with Fisher LSD was used to evaluate the gene expression data; *indicates a significant difference compared to pure scaffolds (1af and 2af) at p < 0.04.

Expression of COL2A1, COL1A2, SOX9 and RUNX2 were quantified after 28 days in culture and the ratios of COL2A1/COL1A2 and SOX9/RUNX2 are presented in Fig. 5. The expression of SOX9/RUNX2 was not significantly different among the scaffolds after 28 days in culture. This was surprising in light of positive staining for type II collagen in some scaffold formulations. SOX9 is an early marker of chondrogenesis^50^ and regulates the expression of downstream targets such as COL2A1^51^,^52^. However, since both SOX9 and RUNX2 are early markers^50^ for chondro- and osteogenesis, respectively, they may have been upregulated and then returned to basal levels by day 28. The expression of COL2A1/COL1A2 was significantly upregulated in CS containing scaffolds (2af-CS and 2rf-CS) compared to pure scaffolds without CS (1af and 2af) (2af-CS, 1af: p = 0.04; 2af-CS, 2af: p = 0.02; 2rf-CS, 1af: p = 0.02; 2rf-CS, 2af: p = 0.01). Varghese *et al*. showed that immobilised CS in hydrogels provided a chondro-favourable environment for condensation and subsequent differentiation of MSCs^53^. However, here unbound CS, which was released quickly (~25% in 3 days), also appeared to promote the differentiation of hBMMSCs down the chondrogenic lineage. Moreover, a higher percentage of pores in CS-containing scaffolds compared to those without CS and type I scaffolds had suitable sizes (50–300 μm) for chondrogenesis. Therefore, it may be that chondrogenic differentiation was driven by a combination of collagen type (type II), the presence of bioactive CS, and physical features of the collagen scaffold itself (although the presence of CS appeared to be the most decisive factor). In short, the highly purified collagen type II may have provided a suitable foundation for the addition of specific factors to promote differentiation. The superiority of collagen type II for chondrogenesis may have implications in designing a scaffold for cartilage repair without the need for sustained delivery of growth factors.

## Methods

### Scaffold fabrication

Type II collagen was extracted from fetal bovine articular cartilage using a limited pepsin digest followed by differential salt fractionation^54^. Type I collagen was extracted from foetal bovine skin using a limited pepsin digest followed by differential salt fractionation^55^,^56^. Chondroitin sulfate A (from bovine trachea) was purchased from Sigma-Aldrich (Poole, Dorset, UK). Type-I and II porous collagenous matrices were prepared as previously described^17^. Briefly, pure afibrillar collagen solutions (type I and II) were produced by adding lyophilised monomers to dilute acetic acid (pH 3.2) to achieve a 0.5% (w/v) solution. The preparations were then homogenised on ice using a hand-held blender (Braun 400 W). Air bubbles were removed by centrifugation (Thermo Electron Corporation IEC CL10 at 5000 rpm for 5 minutes). To produce the afibrillar type II collagen-CS solution (1:1 wt), 0.5 g of CS was dissolved in acetic acid (pH: 3.2) and added drop-wise during homogenisation of the collagen. Air bubbles were removed by vacuum degassing to preserve homogeneity of the suspensions. To induce fibrillogenesis, collagen solutions/suspensions were dialysed at 4 °C for 24 h twice against Tris-buffered saline (50 mM tris-(hydroxymethyl)-aminomethane, 150 mM sodium chloride, pH 7.4). The dialysed monomers were then placed in an incubator at 37 °C for 4 h to allow formation of fibrillar gels. The afibrillar and reconstituted fibrillar suspensions were then washed and frozen (−20 °C) in plastic cylindrical moulds and freeze-dried for 24 h. Scaffold formulations are summarised in Table 1.

### Scaffold characterisation

We characterised 1af scaffolds according to our previously described methods^17^ for comparison to 2af, 2af-CS, 2rf and 2rf-CS conditions. Briefly, compression testing was performed on a Perkin Elmer Dynamic Mechanical Analyzer 7e at a strain rate of 3.5%/min to determine compressive modulus (n = 5). To visualise scaffold microstructure, specimens were cut in transverse and longitudinal sections with a razor, mounted on scanning electron microscopy (SEM) stubs, coated with 4 nm of platinum, and imaged in a JEOL FEG-SEM (JSM-840F) operated at 5 kV. Differences in the thermal diffusion gradient resulting from sample mounting in plastic moulds lead to some degree of pore directionality, creating an anisotropic pore structure, with the orientation of anisotropy in the direction of heat transfer in the mould. Therefore, pore sizes were calculated as area of ellipses (A), yielding a quotient of circularity (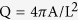, where L is the circumference of the ellipse). To determine solution complex viscosity and confirm fibrillogenesis, rheological measurements were carried out using an Anton Paar 301 rheometer in oscillatory frequency sweeps (angular frequency = 0.1 − 100 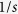, amplitude = 1%, at 20 °C). Porosity was determined by gravimetry, as previously described^57^, and was determined based on scaffold weight and dimensions, and the density of the solid material.

### *In vitro* cell seeding

Scaffolds were sterilised by gamma irradiation at 25 kGy using a Gammacell 1000 (Best Theratronics Ltd., Hertfordshire, UK). Clinical grade bone marrow-derived human MSCs were generated from bone marrow (BM) aspirates collected from the iliac crest of healthy donors. Human samples used in this research project were obtained from the Imperial College Healthcare Tissue Bank (ICHTB). ICHTB is supported by the National Institute for Health Research (NIHR) Biomedical Research Centre based at Imperial College Healthcare NHS Trust and Imperial College London. ICHTB is approved by the UK National Research Ethics Service to release human material for research (12/WA/0196), and the samples for this project were issued from the sub-collection. Informed consent was obtained in accordance with ICHTB requirements and procedures were carried out in accordance with relevant guidelines and regulations. Briefly, 2 ml of BM aspirate was collected in a tube with 100 μl preservative-free heparin. The cells were plated within 24 hours at a density of 10–25 million/636 cm^2^ in alpha modified Eagle’s medium (α-MEM, ThermoFisher Scientific, Paisley, UK), preservative-free heparin (1 UI/ml) (Wockhardt UK Limited, Wrexham, UK) and 5% platelet lysate and then incubated for 3 days under standard conditions (37 °C/5% CO_2_). When cell confluence of 90–100% was achieved, cells were detached with Trypsin-EDTA (0.05% trypsin, 0.53 mM EDTA 4Na) (ThermoFisher Scientific, Paisley, UK) and reseeded at a density of 5000 cells/cm^2^. Cells were cultured in α-MEM without nucleosides supplemented with 10% Foetal Bovine Serum (FBS) (all reagents from Gibco, UK) and used between passages 6 and 8. hBMMSC were immunophenotyped by flow cytometry at passages 1, 4 and 7 and found to be positive for CD90, CD105, CD73, CD44, CD29, CD271, CD56 and MSCA1, and negative for CD34 and CD45. One hundred μl of cell culture medium containing either 450,000 (for histological evaluations) or 1.35 × 10^6^ cells (for all other assays) per scaffold was placed on top of each scaffold in 48-well suspension plates. The use of different cell densities was necessary for optimising assays, but were always accompanied by appropriate controls. Cells were allowed to adhere for 1 h before 500 μl of medium supplemented with 1% antibiotic/antimycotic solution (Sigma-Aldrich, UK) was added. The medium was changed every 2–3 days.

### Assessment of scaffold size change and CS release

As both water and cell-scaffold interactions have the potential to affect scaffold size (swelling/contraction/ECM formation), the diameter of cell-seeded and unseeded scaffolds (both in cell culture media, n = 3) were monitored for 28 days. Plates were imaged by an HP scanner immediately after placing in cell culture media (original diameter, equation (1)), and then after 1 and 28 days. ImageJ was used to measure scaffold diameter. Cell-mediated diameter changes were calculated using equation (1) and normalised to alamarBlue activity (arbitrary unit of mm^2^/fluorescent intensity) to account for scaffold composition-related differences in cell attachment and proliferation. It has been shown that there is a linear relationship between alamarBlue activity and cell number (as determined by DNA quantification^58^) in human periosteum-derived stem cells.


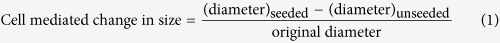


To quantify the amount of CS within scaffolds, scaffolds were digested in a papain solution (2.5 units papain/mL in 5 mM cysteine HCl, 5 mM EDTA in phosphate buffered saline, PBS) for 3 h, and sulfated glycosaminoglycan (GAG) content was determined using a Blyscan Kit (Biocolor, UK), as per the manufacturer’s instructions. To determine the amount of CS released from scaffolds during the first three days of culture, media were collected and CS content was determined, also per the manufacturer’s instructions.

### Assessment of cell viability, morphology and metabolic activity

hBMMSCs viability on scaffolds was assessed by Live/Dead^®^ Cell Viability assay (Molecular Probes^TM^, Invitrogen, UK) after 1 and 14 days in culture. Scaffolds were rinsed twice with PBS and incubated with 4 mM Calcein AM and 4 mM ethidium homodimer-1 in PBS for 15 min, to stain live and dead cells green and red, respectively. Cells were imaged using Zeiss Axiovert 200 inverted fluorescence microscope (Zeiss, Germany) with Carl Zeiss Axiocam Color Microscope Camera.

To assess cell morphology, scaffolds were fixed in 4% (w/v) paraformaldehyde (PFA) for 20 min, washed twice with PBS, and permeabilised with 0.25% (v/v) Triton X-100 in PBS for 30 min. Samples were blocked with 3% (w/v) BSA in PBS for 30 min and the actin cytoskeleton was stained with Alexa Fluor 568 phalloidin (Invitrogen; 1:160) for 1 h. Nuclei were stained with DAPI (Sigma; 1:1000) for 1 min. Samples were then rinsed with PBS and imaged as above.

After 1, 7, 14 and 28 days, scaffolds were transferred to fresh well plates and 500 μl cell culture media containing 10% (v/v) alamarBlue (Invitrogen) was added to each well. Scaffolds were incubated for 4 h before 200 μl of supernatant was transferred to black 96 micro well-plates and read at 544 nm excitation and 590 nm emission.

### Visualisation of cell-mediated ECM formation

Samples were fixed in 4% (w/v) PFA, dehydrated through a graded alcohol series, embedded in paraffin wax and sectioned at 10 μm. Sections were stained with Pico-Sirius Red, Safranin-O, H&E and Alizarin Red using standard protocols, to visualise collagen, sulfated glycosaminoglycans, cells and calcium deposits, respectively.

The cell-mediated formation of collagens type-I and -II were detected with primary monoclonal mouse antibodies against collagen-I (Abcam, UK, ab88288) and collagen-II (Abcam, ab3092) and detected with a goat anti-mouse IgG conjugated to Alexa Fluor 488 (Abcam, ab150117). Primary antibodies were verified to only react with human collagen-I and II (lack of cross-reactivity was checked by staining the 1af and 2af scaffolds without cells). Sections were deparaffinised and rehydrated, and enzymatic antigen retrieval was performed. Briefly, a pepsin equilibration solution (0.02 M HCl) was added to slides for 10 min. Slides were then incubated at 37 °C for 45 min with a pepsin digestion solution (10 mg/ml in 0.02 M HCl) in a humidified chamber before being washed with PBS. Slides were then blocked (5% (v/v) goat serum in 0.01% (v/v) Triton X-100 in PBS) for 60 min, after which slides were incubated at 4 °C overnight with the primary antibody (1:200 for both). Slides were then washed with PBS, incubated for 1 h at room temperature with the secondary antibody (1:200), counterstained with DAPI, mounted and stored at 4 °C in the dark. Slides were imaged with a Zeiss Axiovert 200 inverted fluorescence microscope (Zeiss, Germany) with Carl Zeiss Axiocam Color Microscope Camera.

### Gene expression analysis

After 28 days, total RNA was isolated from scaffolds using an RNeasy kit (Qiagen, Netherlands). Quality of isolated RNA was confirmed by Nanodrop, according to conventional methods. 1 μg of RNA was converted to cDNA by reverse transcription using a QuantiTect Reverse Transcription kit (Qiagen). Real-time quantitative polymerase chain reaction (qPCR) was carried out on a Rotorgene Corbett PCR machine, using the following primer sequences: ß-actin housekeeping gene (forward: AAGCCACCCCACTTCTCTCTAA, reverse: AATGCTATCACCTCCCCTGTGT), SOX9 (forward: AGCGAACGCACATCAAGAC, reverse: CTGTAGGCGATCTGTTGGGG), RUNX2 (forward: TGGTTACTGTCATGGCGGGTA, reverse: TCTCAGATCGTTGAACCTTGCTA), COL2A1 (forward: CCAGATGACCTTCCTACGCC, reverse: TTCAGGGCAGTGTACGTGAAC), COL1A2 (forward: GACATGCTCAGCTTTGTGGA, reverse: CTTTGTCCACGTGGTCCTCT), which were designed using Primer Blast or Primerbank. Expression was quantified using the Brilliant III SYBR Green QPCR Master Mix (Agilent, California, U. S. A.) kit in accordance with the manufacturer’s recommendations. Primers were validated, and their efficiencies were found to be between 0.90 and 1.07. Gene expression levels were normalised to the expression of the housekeeping gene ß-actin and to that in undifferentiated hBMMSC. The results were analyzed using the 2^−ΔΔ*CT*^ method^59^.

### Statistical Analysis

All experimental groups had a sample size of n = 3 for porosity measurements, biochemical, qPCR, and histological analyses, and n = 5 for mechanical property analyses. For pore size analysis, 100 pores per group were examined. Data are presented as mean ± standard deviation. Statistical significance was determined by performing one-way ANOVA for multiple comparisons and post-hoc Tukey test for alamarBlue and Fisher LSD tests for all other experiments with a significance set at the alpha level of 0.05. The actual p value is stated in each case where a significant mean difference was detected. For pore size analysis, since the data was not normally distributed, a nonparametric Mann-Whitney test was performed. The actual p value is stated where there was a significant distribution difference.

## Additional Information

**How to cite this article**: Tamaddon, M. *et al*. Monomeric, porous type II collagen scaffolds promote chondrogenic differentiation of human bone marrow mesenchymal stem cells *in vitro. Sci. Rep.* **7**, 43519; doi: 10.1038/srep43519 (2017).

**Publisher's note:** Springer Nature remains neutral with regard to jurisdictional claims in published maps and institutional affiliations.

## Figures and Tables

**Figure 1:**
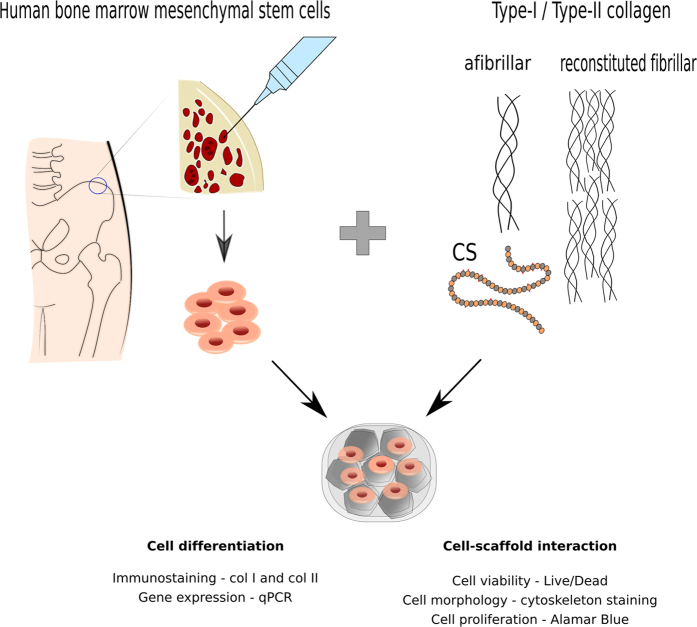
Schematic of experimental procedure: hBMMSCs were isolated from human iliac crest and expanded in culture. Type I and type II collagens were processed to produce afibrillar and reconstituted fibrillar scaffolds incorporating CS. The scaffolds were seeded with hBMMSCs and cell-scaffold interactions including cell viability, morphology and metabolic activity were examined. Cellular differentiation was studied using immunostaining and gene expression.

**Table 1:** Scaffold formulations: type of collagen (type I and II), ultrastructure (afibrillar and reconstituted fibrillar) and the amount of CS.

Collagen type		Collagen:CS weight ratio	Acronym
Type-I	Afibrillar	0	1af
Type-II	Afibrillar	0	2af
		1:1	2af-CS
	Reconstituted fibrillar	0	2rf
		1:1	2rf-CS

**Table 2:** Structural and mechanical characteristics of scaffolds: Q = quotient of circularity of pores (n = 100 pores), percentage of pores having both axes dimensions between 50–300 μm, ɳ* = complex viscosity of collagenous suspension at the frequency of 0.1 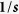, compression modulus presented as mean ± s.d.

	Q	% pores both axes between 50–300 μm	% porosity	Compression modulus (Pa)	ɳ* (Pa.s)
1af	0.79	28%	99.2%	23.7 ± 13.3	1.8
**2af**	0.61*	21%	99.5%	17.1 ± 8.5	0.1
**2af-CS**	0.75	31%	99.7%	286.7 ± 49.1	27.4
**2rf**	0.78	22%	99.4%	26.2 ± 8.4	32.2
**2rf-CS**	0.78	38%	99.7%	55.9 ± 8.2	2.7
